# Ectopic expression of a cyanobacterial flavodoxin in creeping bentgrass impacts plant development and confers broad abiotic stress tolerance

**DOI:** 10.1111/pbi.12638

**Published:** 2016-10-20

**Authors:** Zhigang Li, Shuangrong Yuan, Haiyan Jia, Fangyuan Gao, Man Zhou, Ning Yuan, Peipei Wu, Qian Hu, Dongfa Sun, Hong Luo

**Affiliations:** ^1^ College of Plant Science and TechnologyHuazhong Agricultural UniversityWuhanHubeiChina; ^2^ Department of Genetics and BiochemistryClemson UniversityClemsonSCUSA; ^3^ The Applied Plant Genomics Laboratory of Crop Genomics and Bioinformatics Centreand National Key Laboratory of Crop Genetics and Germplasm EnhancementNanjing Agricultural UniversityNanjingJiangsuChina; ^4^ Crop Research InstituteSichuan Academy of Agricultural SciencesChengduSichuanChina

**Keywords:** cyanobacterial flavodoxin, abiotic stress tolerance, drought tolerance, heat tolerance, methyl viologen resistance, nitrogen starvation, turfgrass, transgenics

## Abstract

Flavodoxin (Fld) plays a pivotal role in photosynthetic microorganisms as an alternative electron carrier flavoprotein under adverse environmental conditions. Cyanobacterial Fld has been demonstrated to be able to substitute ferredoxin of higher plants in most electron transfer processes under stressful conditions. We have explored the potential of Fld for use in improving plant stress response in creeping bentgrass (*Agrostis stolonifera* L.). Overexpression of Fld altered plant growth and development. Most significantly, transgenic plants exhibited drastically enhanced performance under oxidative, drought and heat stress as well as nitrogen (N) starvation, which was associated with higher water retention and cell membrane integrity than wild‐type controls, modified expression of heat‐shock protein genes, production of more reduced thioredoxin, elevated N accumulation and total chlorophyll content as well as up‐regulated expression of nitrite reductase and N transporter genes. Further analysis revealed that the expression of other stress‐related genes was also impacted in Fld‐expressing transgenics. Our data establish a key role of Fld in modulating plant growth and development and plant response to multiple sources of adverse environmental conditions in crop species. This demonstrates the feasibility of manipulating Fld in crop species for genetic engineering of plant stress tolerance.

## Introduction

Abiotic stresses, such as drought, salinity and extreme temperatures, are the major factors impacting plant growth and crop productivity. Most of the adverse environmental conditions inflict damages on the stressed plants through the generation of oxidative and osmotic stresses. Under adverse conditions, plants have evolved numerous mechanisms adapting to diverse environmental challenges to avoid elimination by natural selection. Gene networks involved in plant response to various abiotic stresses including stress perception, signal transduction, production of stress‐related proteins and enzymes, and synthesis of compatible osmolytes and antioxidant metabolites have been extensively studied. Information obtained has been used to develop strategies to genetically engineer crop species improving plant performance under various adverse environmental conditions (Apse and Blumwald, 2002; Flowers, 2004; Mittler, 2006; Seki *et al*., 2003; Vinocur and Altman, 2005; Wang *et al*., 2003; Zhang *et al*., 2004; Zhou and Luo, 2013). Besides molecular strategies manipulating the expression of individual genes for structural and regulatory proteins or noncoding RNA molecules to modify endogenous systems improving plant stress tolerance, alternative approaches based on novel mechanisms derived from microorganisms can also be explored to develop additional avenues of new biotechnology tools for use in plant genetic engineering achieving improved stress tolerance in crop species.

In photo‐microorganisms, substitution of stress‐sensitive enzymes and proteins by resistant isofunctional versions is a typical instance responding to unfriendly environmental conditions. Flavodoxins (Flds), found in some algae and cyanobacteria, are small soluble electron transfer flavoproteins, which participate in many oxido‐reductive processes in prokaryotes and eukaryotes (Blanchard *et al*., 2007; Coba de la Peña *et al*., 2013; Lodeyro *et al*., 2012; Singh *et al*., 2004; Zurbriggen *et al*., 2007). As redox shuttles for essential oxido‐reductive pathways in the stroma, Flds are largely equivalent to those of ferredoxins (Fds), which are ubiquitous small electron transfer proteins, and play a key role in photosynthetic organisms by transferring reducing equivalents produced in the photosynthetic electron transport chain (PETC) to key enzymes including Fd‐NADP reductase (FNR), Fd‐nitrite and sulphite reductases, thioredoxin (Trx)/Fd‐Trx reductase (FTR) and other regulatory and metabolic enzymes involved in critical cellular pathways such as nitrogen (N) and sulphur assimilation, amino acid and fatty acid metabolism, the Calvin cycle, the malate valve and other relevant processes (Balmer *et al*., 2003; Hanke *et al*., 2004; Knaff, 1996; Sétif, 2001; Zurbriggen *et al*., 2008). Moreover, Flds and Fds participate in different routes of cyclic electron flow to prevent stress‐elicited excessive reducing power in the PETC and the stroma (Kramer *et al*., 2004; Munekage *et al*., 2004), maintaining a balanced Fd redox state, which also plays a role in the intracellular signalling pathway between chloroplast and nucleus (Knaff, 1996). Given their importance as the key proteins in major metabolic pathways crucial for cell function, impaired activities of Fds would negatively impact plant growth and productivity (Holtgrefe *et al*., 2003). Unfortunately, Fds are extremely sensitive to environmental stress and their expression is down‐regulated transcriptionally and post‐transcriptionally by numerous adverse environmental conditions (Holtgrefe *et al*., 2003; Petrack *et al*., 1998; Tognetti *et al*., 2006; Zurbriggen *et al*., 2008).

In photosynthetic microorganisms, the Fd protein is the preferred electron carrier under normal conditions, whereas the *Fld* gene is typically induced under environmental or nutritional hardships (Singh *et al*., 2004; Yousef *et al*., 2003; Zheng *et al*., 1999). Overexpression of Fld in *Escherichia coli* enhanced bacterial oxidative stress (Coba de la Pena *et al*., 2013; Zheng *et al*., 1999). In higher plants, Fd retention in the plant lineage is probably related to its higher efficiency as an electron carrier, compared with Fld, which was lost along with evolution (reviewed by Karlusich *et al*., 2014). However, *in vitro* study has demonstrated that cyanobacterial Fld is still able to function as electron carrier efficiently interacting with Fd‐dependent plant partner enzymes, such as chloroplast FNR (Nogués *et al*., 2004). Transgenic (TG) studies in tobacco show that Fld is able to complement Fd deficiency in knocked‐down TG plants (Blanco *et al*., 2011). Further study exploring the potential use of bacterial Fld in higher plants for their protection from adverse environmental conditions revealed that TG tobacco expressing Fld exhibits significantly improved plant resistance to various adverse environmental conditions including oxidative stress, high light intensities, chilling, UV radiation, phytotoxicity, iron deficiency and water deficit (Ceccoli *et al*., 2011; 2012; Tognetti *et al*., 2006, 2007a,b). Fld‐expressing TG *Medicago truncatula* plants also exhibited less‐affected N fixation in nodules by salt stress than in wild‐type (WT) control plants (Coba de la Peña *et al*., 2010). This strongly suggests that introduction of Fld into plants could serve as a new strategy for genetic engineering of plant stress tolerance. So far, however, the feasibility of using Fld in agriculturally and economically important crop species, especially in perennial crops, for enhancing plant stress tolerance has not been extensively explored, and the molecular mechanisms underlying Fld‐mediated plant stress response and plant development also remain elusive. In this study, we generated TG creeping bentgrass (*Agrostis stolonifera* L.) plants ectopically expressing Fld and conducted further analysis of the TG turfgrass to investigate the role Fld plays in controlling plant development and plant response to environmental stress. Using TG approach to study the impact of Fld in an important crop species, we attempt to address the following questions. Can Fld function in perennial crops to impact plant growth? Is Fld implicated in plant stress response that contributes to enhancing plant tolerance to various abiotic stresses in grasses? And if so, what is the molecular mechanism underlying Fld‐mediated plant stress response in perennial grasses?

## Results

### Generation and molecular analysis of TG plants expressing the cyanobacterial *Fld* gene

To explore the effectiveness of a cyanobacterial Fld in perennial grasses for improving plant response to environmental stresses, we first synthesized a 669‐bp DNA fragment containing the coding sequence of a pea FNR chloroplast‐targeting transit signal peptide (Newman and Gray, 1988; Serra *et al*., 1995) translationally fused to the cyanobacterial *Fld* gene (Fig. S1a). FNR transit peptide serves to target the fusion protein into the chloroplast. The *FNR‐Fld* gene was then used to prepare a chimeric gene construct, pUbi:*FNR‐Fld*/p35S:*bar* (Fig. S1b). In this construct, the *FNR‐Fld* gene under the control of the corn ubiquitin (Ubi) promoter was linked to the herbicide glufosinate (phosphinothricin) resistance gene, *bar*, which was driven by the cauliflower mosaic virus 35S (CaMV35S) promoter. The construct was introduced into the creeping bentgrass cultivar, Penn A‐4, to produce a total of 28 independent TG lines. RT‐PCR analysis and Northern hybridization using *Fld* gene as a probe demonstrated *Fld* expression in all TG lines (see examples in Fig. S1c). Real‐time PCR analysis further confirmed the high‐level expression of *Fld* in transgenics (see examples in Fig. S1d). When grown and evaluated in glasshouse, the TG lines were all similar to each other in morphology, development and response to various cultivation conditions. Seven TG lines, TG4, TG5, TG6, TG16, TG17, TG23 and TG24, were selected and clonally multiplied by vegetative propagation for further analysis.

### Overexpression of Fld leads to modified plant growth and development

**Figure 1:**
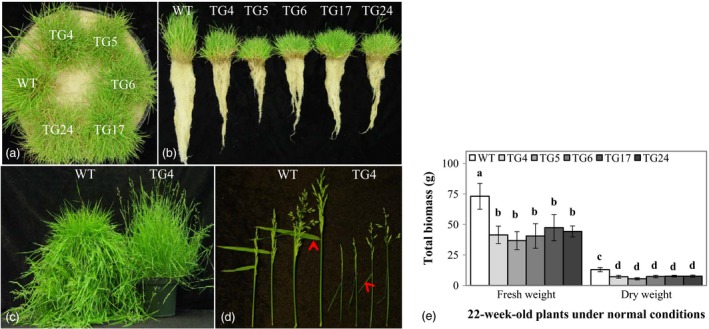
Overexpression of Fld leads to modified plant growth and development in transgenic (TG) creeping bentgrass. Wild‐type (WT) and five TG plants developed in pure sand under normal conditions in a growth room for 22 weeks (a) were carefully removed from the Elite 1200 pots and washed briefly to display their root development (b). (c) The TG plants exhibited a different phenotype from WT controls at the reproductive stage. (d) The TG inflorescence was different from that of the WT controls in spike size and flag leaf angle. The leaf angle between the spikelet stem and the midrib of the ventral side of the flag leaf was indicated by arrows. (e) Total biomass (fresh and dry weights) of the 22‐week‐old TG and WT plants. The statistically significant difference between groups was determined by one‐way ANOVA. Means not sharing the same letter are statistically significantly different (*P* < 0.05).

As shown in Figure 1a–c, Fld‐expressing TG plants exhibited significant difference from WT controls. Overexpression of Fld caused dramatic change in plant morphology, resulting in retarded plant growth and development. When comparing plants maintained in Elite 1200 pots with pure sand for 22 weeks, the TG plants grew significantly slower than WT controls, with the total biomass being an average of 44.7% less in fresh weight and an average of 40.9% less in dry weight, respectively (Figure 1e). However, the TG plants exhibited significantly higher tillering rate than WT controls (Fig. S2a). Upon vernalization, both WT and TG plants flowered normally (Figure 1c). However, the TG plants produced smaller inflorescence with less branches and spikelets than WT controls (Figure 1d, Fig. S2b). Interestingly, the flag leaf of each inflorescence in TG plants was much more open than that of the WT controls, with a leaf–stem angle of more than 90° (Figure 1d). Taken together, cyanobacterial Fld impacted plant development in both vegetative and reproductive stages, causing changes in plant morphology and delayed plant growth in TG creeping bentgrass.

### Overexpression of Fld improves plant oxidative stress tolerance

**Figure 2:**
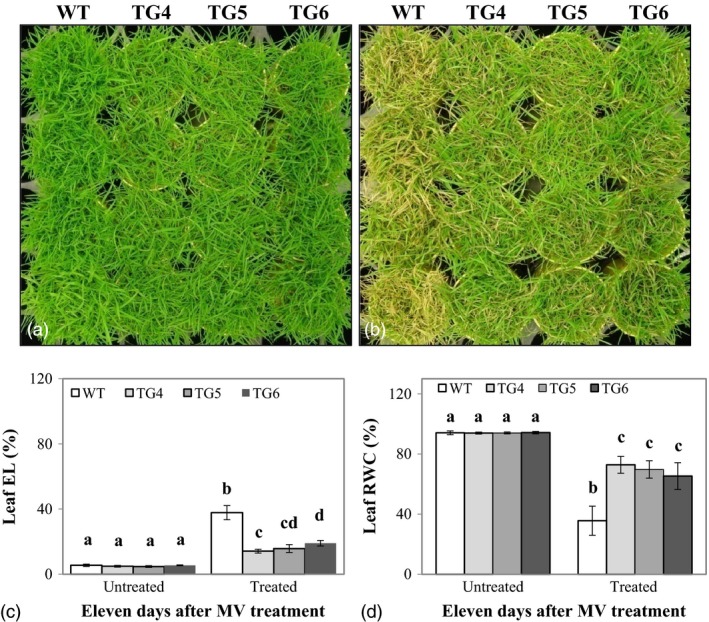
Transgenic (TG) turfgrass overexpressing flavodoxin (Fld) exhibits enhanced oxidative stress tolerance compared to wild‐type (WT) controls. TG and WT plants were repotted in cone‐tainers and grown for 10 weeks under normal maintenance (a). Fully developed plants were sprayed daily with redox‐cycling herbicide and methyl viologen (MV, 30 μm with 0.02% Triton X‐100) for 3 days. The TG plants exhibited enhanced resistance to MV (b) with significantly lower leaf electrolyte leakage (EL) (c), but higher relative water content (RWC) (d) than WT controls. Photographs were taken before (a) and 4 days after the 3‐day MV treatments (b). The statistically significant difference in leaf EL and RWC between groups was determined by one‐way ANOVA. Means not sharing the same letter are statistically significantly different (*P* < 0.05).

To investigate how Fld expression in TG plants would impact plant response to environmental stress, we first set to examine performance of the Fld‐overexpressing TG lines grown under oxidative stress. Eight‐week‐old plants were treated with 30 μm of methyl viologen (MV, Sigma‐Aldrich Co. LLC, MO), an artificial acceptor and donor which accepts electrons from photosystem I and transfers them to molecular oxygen to produce reactive oxygen species (ROS, Semenov *et al*., 2003). As illustrated in Figure 2b for plants grown in cone‐tainers and subjected to MV treatment for 3 days, Fld‐expressing transgenics showed significantly less damage than the non‐TG control plants without Fld. The difference in MV‐elicited damage between TG and WT control plants became more pronounced with the increasing treatment times (Fig. S3a). Similar results were also observed for plants grown together in big pots (Fig. S3b).

Upon a 3‐day MV treatment, the plants were allowed to recover by sufficient watering for 10 days, and the shoots were clipped for use in measuring EL and RWC. As shown in Figure 2c, d, although no differences were observed between the TG and WT control plants for both leaf EL and RWC under normal growth conditions, the leaf EL of the MV‐treated TG plants was significantly lower than that of the MV‐treated WT controls (Figure 2c), and the leaf water loss in the MV‐treated Fld‐expressing plants was also significantly less than that in the MV‐treated control plants without Fld (Figure 2d), suggesting that under MV treatment, transgenics exhibited less cell membrane damage and enhanced water retention capacity.

### Overexpression of Fld results in enhanced drought tolerance in TG plants

**Figure 3:**
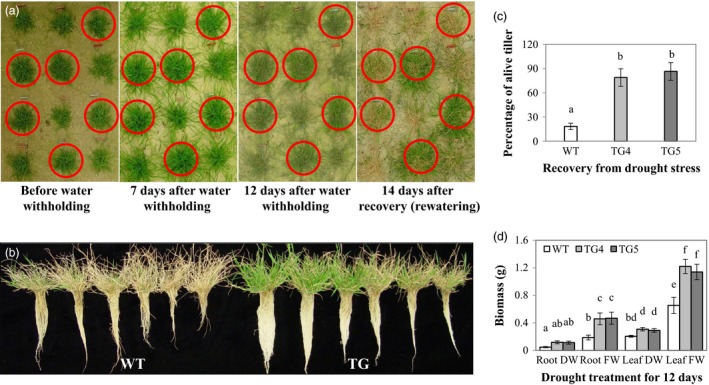
Transgenic (TG) turfgrass overexpressing flavodoxin (Fld) exhibits enhanced drought tolerance compared to wild‐type (WT) controls. Individual TG and WT plants originated from five tillers were repotted randomly in a tray (50 × 35 × 10 cm) and developed for 40 days under normal growth conditions (a). The plants were then treated by water withholding for 12 days until plants were heavily suffered, followed by recovery with sufficient watering for 12 days (a). The TG plants exhibited much higher tolerance to drought stress under water deprivation conditions and recovered much faster after rewatering than WT controls (a, b). The percentage of the survived tillers in TG plants was significantly higher than that in WT controls (c). Similar results were also obtained for plant biomass. TG plants exhibited significantly higher biomass than WT controls (d). Photographs were taken before and during water withholding and after recovery. TG plants were circled in red. The figure shows results from two representative TG lines (TG4 and TG5). The statistically significant difference in percentage of the survived tillers and biomass between groups was determined by one‐way ANOVA. Means not sharing the same letter are statistically significantly different (*P* < 0.05).

To examine how ectopic expression of the Fld in creeping bentgrass would impact plant response to water stress, we evaluated the performance of the Fld‐overexpressing TG lines grown in sand under drought conditions. The results indicated that compared to WT controls, the TG lines tested all exhibited significantly enhanced drought tolerance. As exemplified in Figure 3a for line TG4, individual Fld‐expressing TG plants (circled in red) and WT controls developed from five tillers were randomly planted together in a tray (50 × 35 × 10 cm) filled with sand. Forty days after development under normal growth conditions, drought stress was applied by water withholding for 12 days until plants were heavily suffered from water deficiency. Plants were then allowed to recover by sufficient watering for 14 days. As shown in Figure 3a–c, the TG plants were all recovered from the drought‐elicited damage with more than 80% of the tillers alive, whereas almost all the WT control plants died. Moreover, the TG roots and shoots displayed significantly less growth inhibition than WT controls under drought stress conditions. As a result, TG plants produced more biomass than WT controls as reflected by significantly higher fresh weights of roots and shoots in TG plants than in WT controls (Figure 3d). Similar results of enhanced drought tolerance in Fld transgenics were also obtained in various TG lines tested in cone‐tainers (Fig. S4).

### Overexpression of Fld increases plant heat tolerance that is associated with modified expression of heat‐shock protein (HSP) genes and the production of more reduced Trx

**Figure 4:**
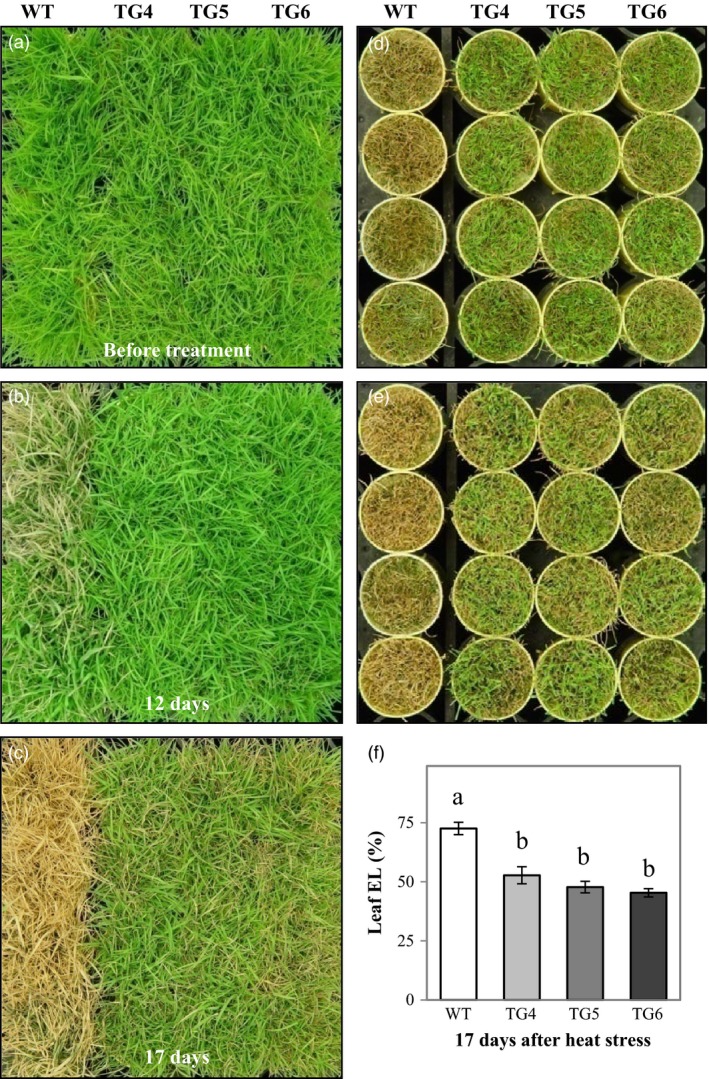
Overexpression of flavodoxin (Fld) enhances creeping bentgrass heat stress tolerance. Transgenic (TG) lines (TG4, 5 and 6) and wild‐type (WT) control plants were repotted in cone‐tainers and arranged in hexagon shape. After 10‐week full development under normal maintenance (a), WT and TG plants were exposed to 35 °C/40 °C (night/daytime) treatment regime for 17 days (b, c). The TG lines showed significantly enhanced heat tolerance compared to WT controls (b, c). After heat stress, shoots were clipped (d) for electrolyte leakage (EL) measurement. The shoot recovery of TG plants was much better than that of the WT controls after clipping and recovery for 1 week (e). The EL of the WT plants was significantly higher than that of the three independent TG lines (f). Photographs were taken on indicated dates. The statistically significant difference in leaf EL between groups was determined by one‐way ANOVA. Means not sharing the same letter are statistically significantly different (*P* < 0.05).

To investigate whether or not ectopic expression of the cyanobacterial Fld would impact plant thermotolerance, we evaluated the performance of the Fld‐overexpressing TG plants under heat stress. As demonstrated in Figure 4, the TG plants grown in cone‐tainers outperformed WT controls under heat stress conditions (Figure 4b–e). While the control plants without Fld became severely damaged (carbohydrate deprivation) 17 days after heat exposure, the Fld‐expressing TG plants only exhibited minor heat‐inflicted symptom (Figure 4b–e). All the TG plants tested recovered upon release from the stress, whereas most of the WT controls did not survive the treatment (Figure 4c, e). The enhanced heat tolerance observed in the TG plants was associated with a lower leaf cell EL than the WT controls under the heat stress (Figure 4f), suggesting an enhanced cell membrane integrity in Fld‐expressing TG plants compared to the non‐TG WT controls.

**Figure 5:**
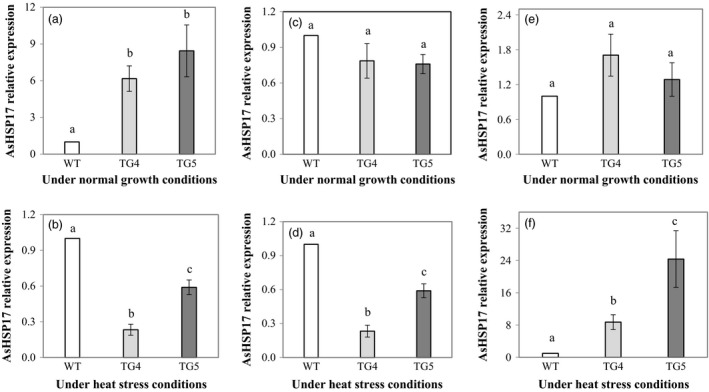
Expression profiles of three heat‐shock protein (HSP) genes, *AsHSP17* (a, b), *AsHSP26.7* (c, d) and *AsHSP26.8* (e, f), in transgenic (TG) and wild‐type (WT) control plants under normal growth and heat stress conditions. ΔΔCt method was used for real‐time RT‐PCR analysis. Two reference genes, *AsACT1* and *AsUBQ*, were used as endogenous controls and showed similar results. The data presented are those using *AsUBQ* as the endogenous controls (Zhou *et al*., 2013). Three biological replicates and three technical replicates were used for statistic analysis. Error bars represent SE (n = 9). The statistically significant difference between groups was determined by one‐way ANOVA. Means not sharing the same letter are statistically significantly different (*P* < 0.05).

To better understand the molecular mechanism of Fld‐mediated plant heat tolerance, we analysed the expression of the three creeping bentgrass small *HSP* (*sHSP*) genes, *AsHSP17* (Sun *et al*., 2016), *AsHSP26.7* and *AsHSP26.8* (Wang and Luthe, 2003) with molecular weight of 17, 26.7 and 26.8 kD, respectively. The results showed that under normal growth conditions, the expression of the three *sHSP* genes was all extremely low in both the TG plants and WT controls (Fig. S5), and no significant difference was observed between the TG plants and WT controls for *AsHSP26.7* and *AsHSP26.8* (Figure 5c, e). However, the expression of the *AsHSP17* in the TG plants was significantly higher than that in WT controls (Figure 5a). Upon heat stress (40 °C for 4 h), the three *AsHSP* genes in both WT and the TG plants were all significantly induced. Compared to the normal growth conditions, the heat stress‐induced expression of the *AsHSP17*,* AsHSP26.7* and *AsHSP26.8* increased 3.8 × 10^4^, 2.8 × 10^5^ and 350 times, respectively, in WT controls, and 1300, 3.7 × 10^4^ and 3500 times, respectively, in the Fld transgenics. The expression of the *AsHSP17.0* and *AsHSP26.7* was significantly lower (Figure 5b, d), whereas that of the *AsHSP26.8* was significantly higher (Figure 5f), in the TG plants than in WT controls.

**Figure 6:**
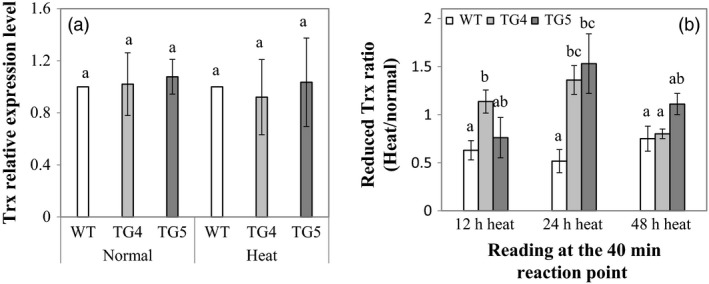
Thioredoxin (*Trx*) gene expression and reduced Trx content in transgenic (TG) and wild‐type (WT) control plants. (a) Expression of the *Trx* gene, *Trxh* in TG and WT control plants under normal and heat stress conditions. ΔΔCt method was used for real‐time RT‐PCR analysis. Two reference genes, *AsACT1* and *AsUBQ*, were used as endogenous controls and showed similar results. The data presented are those using *AsUBQ* as the endogenous control (Zhou *et al*., 2013). Three biological replicates and three technical replicates were used for statistic analysis. Error bars represent SE (n = 9). (b) Ratio of the reduced Trx contents under heat stress and normal conditions in TG and WT control plants. The statistically significant difference between groups was determined by one‐way ANOVA. Means not sharing the same letter are statistically significantly different (*P* < 0.05).

To investigate what role Fld plays in plant redox shuttling under stress conditions, we conducted tests to compare reduced Trx contents between the Fld‐expressing transgenics and WT controls under normal and heat stress conditions. As illustrated in Figure 6, although no significant difference was observed between the TG plants and WT controls in the expression of one of the *Trx* gene, *AsTrx h* (Buchanan, 2016), under both normal and heat stress conditions (Figure 6a), there was more reduced Trx produced in the TG plants than in WT controls under heat treatments, especially under prolonged heat stress (Figure 6b).

### Fld transgenics exhibit enhanced tolerance to N starvation associated with elevated N accumulation and total chlorophyll content as well as up‐regulated expression of NiR and N transporter genes

**Figure 7:**
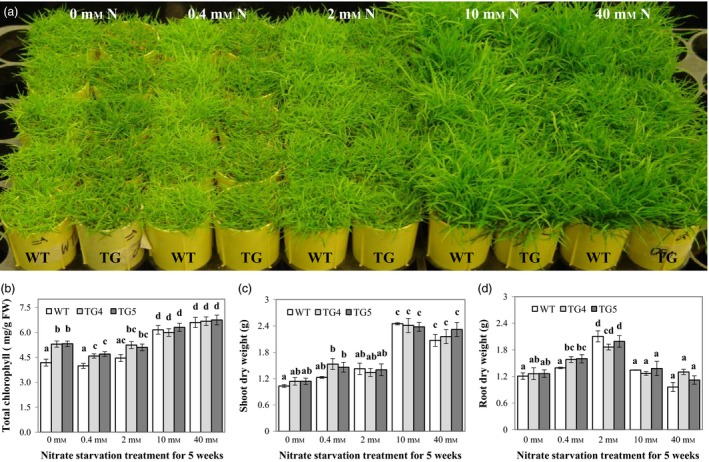
Flavodoxin (Fld)‐expressing transgenic (TG) plants exhibit enhanced tolerance to nitrogen (N) starvation. (a) Fully developed TG and wild‐type (WT) plants in cone‐tainers were maintained under normal growth conditions for 9 weeks and then treated with 1× MS medium containing 0, 0.4, 2, 10 or 40 mm nitrate (15 mL per day) for 5 weeks. Leaf total chlorophyll content was measured for both TG and WT control plants (b). Shoot and root tissues (n = 5) were harvested and processed for dry weight (c, d) measurement. TG4 was shown as a representative TG line for plant response to N starvation. The statistically significant difference in total chlorophyll, shoot and root dry weights between groups was determined by one‐way ANOVA. Means not sharing the same letter are statistically significantly different (*P* < 0.05).

N is an essential nutrient for plant growth and development. A number of plant regulatory and metabolic enzymes, such as Fd‐NiR that catalyses the reduction of nitrite to ammonia, are involved in N assimilation and metabolism, and many of them use Fd or Fld as electron donor (Arizmendi and Serra, 1990; Zurbriggen *et al*., 2008). This prompted us to investigate the potential role Fld may play in plant adaptation to N starvation and plant N assimilation. To this end, we compared plant growth under various N concentrations and measured relevant biochemical, physiological and molecular parameters in both TG and WT control plants. As demonstrated in Figure 7a for plants subjected to a 5‐week‐long treatment with different N supplies, the Fld‐expressing TG plants displayed greener shoot colour than WT controls under N starvation conditions (0, 0.4 and 2 mm). Further analysis revealed that compared to N‐sufficient (10 mm) or N‐excessive plants (40 mm), plants grown under N deficiency conditions (0, 0.4 and 2 mm) had reduced total chlorophyll contents (Figure 7b). While the total chlorophyll contents were similar between the TG plants and WT controls under N‐sufficient and N‐excessive conditions, a significantly higher chlorophyll content was observed in the TG plants than in WT controls under N‐starved conditions (Figure 7b), suggesting that the TG plants may be less prone to chlorophyll degradation and, therefore, likely maintain a higher capacity in photosynthesis than WT controls under N starvation conditions. Moreover, shoot and root dry weights of the TG plants were higher than those of the WT controls 5 weeks after 0.4 mm N treatment although this difference was statistically insignificant in shoot dry weight (Figure 7c, d). Interestingly, suboptimum N supplies inhibited shoot growth, but appeared to promote root growth in both the TG and WT control plants (Figure 7c, d, Fig. S6). It is also noteworthy that over‐fertilization (40 mm N concentration) appeared to inhibit plant growth as reflected by the reduced shoot and root biomass compared to plants under optimum N supplies (Figure 7c, d, Fig. S6). This inhibition in WT controls was greater than that in the TG plants although the difference was statistically insignificant (Figure 7c, d). Taken together, these results indicated that overexpression of Fld positively impacted plant response to N starvation and may have contributed to maintaining plant photosynthesis under N deficiency conditions.

**Figure 8:**
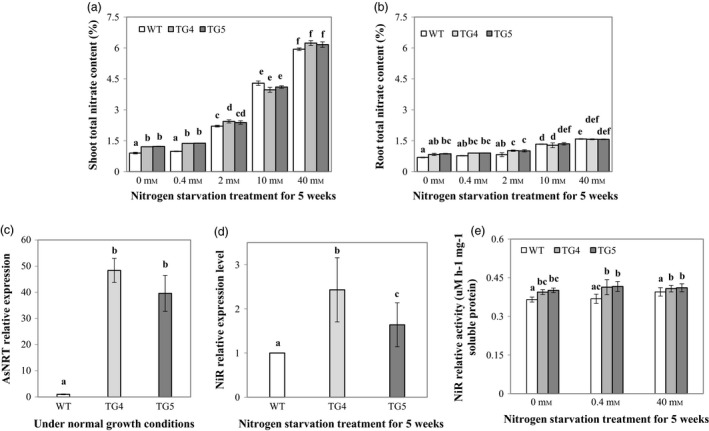
The impact of flavodoxin (Fld) on plant nitrogen (N) uptake and assimilation. (a) Shoot total N content (n = 4) of wild‐type (WT) and transgenic (TG) plants measured 5 weeks after application of different N concentrations. (b) Root total N content (n = 4) of WT and TG plants measured 5 weeks after application of different N concentrations. (c) Expression of the N transporter *AsNRT* in TG and WT control plants under normal growth conditions. (d) Expression of the nitrate reductase (*NiR*) gene in TG and WT control plants 5 weeks after N starvation. (e) NiR activity in TG and WT control plants under normal and N starvation conditions. The statistically significant difference between groups was determined by one‐way ANOVA. Means not sharing the same letter are statistically significantly different (*P* < 0.05).

To further understand what caused the differential growth between the Fld‐expressing transgenics and WT control plants under N deprivation conditions, we measured shoot and root total N contents in the TG and WT control plants under various N supplies (0, 0.4, 2, 10 and 40 mm). The results indicated that the higher the concentration of the N solution applied, the more the total N amount that plants accumulated in shoots and roots (Figure 8a, b). This tendency in N accumulation was more pronounced in shoot than in root. TG shoots and roots accumulated more N than WT controls under N‐starved conditions, and the differences in N accumulation between TG and WT control plants were mostly significant (0, 0.4 and 2 mm, Figure 8a, b), suggesting an enhanced N use efficiency (NUE) in TG plants. To investigate what caused the enhanced NUE in Fld transgenics, we examined the transcript levels of the genes encoding a high affinity nitrate transporter and the NiR, the key enzymes in N assimilation pathway. As shown in Figure 8c, the expression of the nitrite transporter (Kotur *et al*., 2013) gene, *AsNRT*, was significantly up‐regulated in TG plants in comparison with WT controls. Further analysis revealed that there was no significant difference in *AsNiR* (KR911829) expression between TG and WT control plants (Figure 8d). However, the enzyme activity of the NiR was significantly higher in the TG plants than in WT controls under N starvation conditions (Figure 8e).

### The impact of Fld on the expression of other stress‐related genes

**Figure 9:**
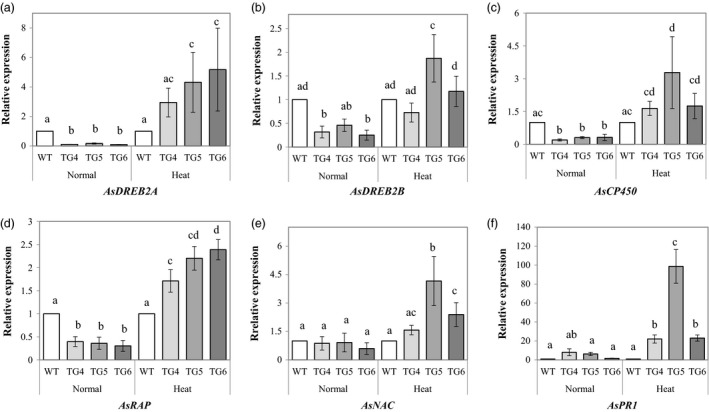
Expression profiles of six stress‐related genes, *AsDREB2A* (a), *AsDREB2B* (b), *AsCP450* (c), *AsRAP* (d), *AsNAC* (e) and *AsPR1* (f) in transgenic (TG) and wild‐type (WT) control plants under normal growth and heat stress conditions. ΔΔCt method was used for real‐time RT‐PCR analysis. Two reference genes, As*ACT1* and *AsUBQ*, were used as the internal controls (Zhou *et al*., 2013). Three biological replicates and three technical replicates were used for statistic analysis. Error bars indicate SE (n = 9). The statistically significant difference between groups was determined by one‐way ANOVA. Means not sharing the same letter are statistically significantly different (*P* < 0.05).

To examine how Fld affects other stress‐related genes in TG plants, we cloned partial sequences of six creeping bentgrass genes encoding AsDREB2A (dehydration response element binding protein, 2A), AsDREB2B (dehydration response element binding protein, 2B) (Matsukura *et al*., 2010; Mizoi *et al*., 2013), AsCP450 (cytochrome P450 94 family‐like) (Aubert *et al*., 2015), AsRAP (ethylene response transcription factor) (Hinz *et al*., 2010), AsNAC (NAC domain protein) (Olsen *et al*., 2005; Yao *et al*., 2012; Zhou *et al*., 2013) and AsPR1 (plant pathogenesis‐related protein) (Sels *et al*., 2008) that are highly homologous to their counterparts in *Brachypodium distachyon*. Gene expression analysis using real‐time RT‐PCR analyses demonstrated that under normal growth conditions, the expression of the *AsDREB2A* (Figure 9a), *AsDREB2B* (Figure 9b) and *AsCP450* (Figure 9c) all went down sharply in the Fld‐expressing TG plants compared to WT controls. However, the expression of these genes in the Fld TG plants was significantly up‐regulated upon heat stress, showing no significant difference from, or higher than that of the WT controls (Figure 9a–c). The expression of an ethylene response transcription factor (*ERF*) gene, *AsRAP*, was also down‐regulated in the Fld TG plants under normal growth conditions, but significantly up‐regulated upon heat stress compared to WT controls (Figure 9d). Another stress‐related transcription factor gene, *AsNAC*, of the Fld TG plants showed no difference in expression from that of the WT controls under normal growth conditions (Figure 9e, left panel). However, *AsNAC* expression was significantly induced in the majority of the TG plants upon heat stress and significantly higher than that of the WT controls (Figure 9e, right panel). Similarly, *AsPR1* expression in the Fld TG plants showed no difference from that in WT controls under normal conditions, but was markedly induced and significantly higher than that in WT controls upon heat stress (Figure 9f). Taken together, these data demonstrated that Fld overexpression resulted in enhanced expression of a number of important stress‐related genes in TG creeping bentgrass plants under stressful conditions.

## Discussion

Chloroplast Fd, a mobile electron carrier to distribute reducing equivalents generated in the PETC during photosynthesis to various essential metabolic, regulatory and dissipative pathways, is critical in the physiology of the plant cell. The ubiquitous small electron transfer protein delivers electrons to key enzymes including FNR, Fd‐nitrite and sulphite reductases, gluoxoglutarate aminotransferase, fatty acid desaturase and FTR (Balmer *et al*., 2003; Hanke *et al*., 2004; Knaff, 1996; Sétif, 2001; Zurbriggen *et al*., 2008). As such, the steady supply of reduced Fd in plant cell is essential for the function and regulation of many important cellular pathways and metabolisms including carbon fixation and allocation, N and sulphur assimilation, amino acid synthesis and fatty acid desaturation. This multiplicity of functions of Fd in plants suggests its potential role in various biological processes critical for plant development and plant response to environmental cues. Indeed, diminished leaf Fd content by antisense suppression of the *Fd* gene expression in TG potato plants altered electron distribution and photosynthesis, resulting in a pleiotropic phenotype in TG plants such as lower CO_2_ assimilation rates, progressive loss of chlorophyll in a dynamic process, and therefore pale green or yellowish leaves over time, perturbed distribution of electrons, lower Trx reduction and photoinhibition and decreased growth rates (Holtgrefe *et al*., 2003).

Fld plays a vital role in photosynthetic microorganisms as an alternative electron carrier flavoprotein under adverse environmental conditions. Cyanobacterial Fld can efficiently substitute Fd of higher plants in most electron transfer processes under stressful conditions and has been demonstrated in TG tobacco plants conferring increased tolerance to different abiotic stresses (Tognetti *et al*., 2006, 2007a,b; Zurbriggen *et al*., 2008). However, Fld‐mediated improvement in plant stress response was not observed in *M. truncatula* although compared to WT controls, Fld‐expressing TG *M. truncatula* plants exhibited less‐affected N fixation in nodules by salt stress (Coba de la Peña *et al*., 2010). In this study, we investigated the impact of Fld on creeping bentgrass, an important perennial crop species. Our data revealed that overexpression of Fld in creeping bentgrass resulted in altered plant growth and development, and most significantly, Fld‐expressing transgenics exhibited enhanced tolerance to multiple sources of adverse environmental conditions, including oxidative stress, water deficit, heat stress and N starvation. Our results suggest that like in model species, tobacco, Fld is also functional in perennial crop plants to compensate for the decline and impaired operation of Fd in the chloroplast of plants subjected to stressful conditions. It should be noted that FNR transit peptide has previously been demonstrated to efficiently target the FNR‐Fld fusion protein into the tobacco chloroplast. In TG plants harbouring *FNR‐Fld* fusion gene, Fld was successfully targeted to chloroplast, and the TG tobacco exhibited enhanced abiotic stress tolerance compared with WT controls and the TG plants harbouring *Fld* gene alone, in which, Fld was only cytoplasm‐localized (Tognetti *et al*., 2006). The same strategy used in our study proves to be successful. TG creeping bentgrass produced expresses *FNR‐Fld* fusion gene and exhibits altered plant growth, development and response to abiotic stress in comparison with non‐TG WT control plants.

In this study, Fld transgenics exhibited significantly altered plant development under normal growth conditions such as reduced biomass, delayed plant growth and altered inflorescence (Figure 1). Sakakibara *et al*. (2012) studied the protein–protein interaction of Fd and NiR by NMR spectroscopy and found that although Fds from higher plant (maize) and cyanobacterium (*Leptolyngbya boryana*) share high structural similarities, they differ significantly in the interaction with cyanobacterial NiR, highlighting the different molecular interaction between Fd and partner enzyme. Similarly, we speculate that in Fld TG creeping bentgrass, both Fld and Fd bind to partner enzymes under normal growth conditions, but the different interactions of these two flavoproteins with the same partners may alter the way the reducing equivalents generated in the PETC are distributed to various essential metabolic, regulatory and dissipative pathways therefore impacting plant growth and development.

In photosynthesizing chloroplast, rapid transients of photon capture, electron fluxes and redox potentials cause ROS to be released. As has previously been demonstrated that in Fld‐expressing TG tobacco plants, the introduced flavoprotein exhibited antioxidant activity under stressed conditions with lower ROS accumulation (Tognetti *et al*., 2006). The Fld‐mediated ROS dissipation and scavenging resulted in reduced oxidative damage to sensitive enzymes, membranes, pigments and photosynthesis (Tognetti *et al*., 2006). As the toxic by‐products of aerobic metabolism, ROS also functions as one of a network of diverse signals. ROS signalling is a central component of the retrograde signalling network from the photosynthesizing chloroplast to the cytosol, mitochondrion and nucleus (Chan *et al*., 2016; Dietz, 2015). As key members in the complex signalling network of cells, ROS play a multitude of signalling roles in different organisms (D'Autréaux and Toledano, 2007; Dietz *et al*., 2016; Mittler *et al*., 2011). Hence, it is conceivable that overexpression of Fld in TG creeping bentgrass may have changed cell ROS homeostasis, which probably impacted related ROS‐mediated signalling pathways, resulting in altered plant growth and development compared to WT controls (Figure 1).

It is noteworthy that Fld‐mediated significant change in plant development in TG creeping bentgrass (Figure 1) was not observed in Fld‐expressing tobacco plants (Tognetti *et al*., 2006, 2007a,b; Zurbriggen *et al*., 2008). These contrasting Fld effects on plant growth may imply a potentially differential regulation of the ROS signalling between dicot and monocot plant species. The regulation of plant growth and development in evolutionarily advanced monocot species may be more fine‐tuned than that in dicot species therefore prone to being impacted by any altered regulation machinery. It would be interesting to find out whether the similar phenomenon could also be observed in other monocot crops, and we are currently conducting research in rice to test this hypothesis.

Consistent with the observations in Fld‐expressing TG tobacco plants (Tognetti *et al*., 2006, 2007a,b; Zurbriggen *et al*., 2008), the Fld‐expressing TG creeping bentgrass also exhibited enhanced tolerance to multiple adverse environmental conditions including oxidative, heat and drought stress and N starvation. The enhanced stress tolerance mediated by Fld was associated with changes in various factors known to be involved in plant stress response. For instance, sHSPs present in virtually all organisms are stress‐induced molecular chaperones. They bind and stabilize their client proteins that have become denatured under stress conditions. Most of them are highly up‐regulated in response to heat and have a clear role in thermotolerance (Atkinson and Urwin, 2012; Merino *et al*., 2014; Sun *et al*., 2016; Waters, 2012). Three *AsHSP* genes examined in this study, *AsHSP17* (Sun *et al*., 2016), *AsHSP26.7* and *AsHSP26.8* (Wang and Luthe, 2003), were all significantly induced, but differentially regulated in WT and the TG plants (Figure 5), suggesting Fld implication in triggering one of the important stress response mechanisms, contributing to an enhanced plant tolerance to heat.

Trx, a class of small, ubiquitous redox proteins, plays a central role in various important biological processes, including distribution of reducing equivalents generated during photosynthesis. In algae, Fd has been proven to be a more efficient electron carrier than Fld in most reactions assayed *in vitro*, including Trx reduction by FTR in reconstituted systems, and a strongly preferred electron donor over Fld for nitrate reduction via nitrite reductase (NiR) and glutamine synthetase (Meimberg and Mühlenhoff, 1999; Vigara *et al*., 1998). Our study indicates that overexpression of Fld in the TG creeping bentgrass led to an enhanced electron delivery efficiency in the PETC, producing more reduced Trx beneficial to plant stress response (Figure 6b).

Fld‐mediated resistance to multiple stresses in a perennial grass species further confirmed the versatility of Fld in being fully operational in plant cell as an alternative intermediate for the PETC, strongly suggesting that under harsh environmental conditions, Fld could replace Fd in many, if not all, chloroplast‐based pathways to maintain important metabolism within the cell and protect plants from damages elicited by the adverse environmental conditions. It should be noted that as observed in Fld‐expressing *M. truncatula* (Coba de la Peña *et al*., 2010), Fld‐expressing creeping bentgrass did not show significant difference from WT controls in plant response to salinity stress (Fig. S7), indicating that Fld is ineffective in prevention of all the alterations produced by oxidative damage in plants. The salt‐triggered ROS might induce both oxidative and toxic damages that Fld may not be able to counteract.

Taking together, the broad‐range stress tolerance exhibited in TG creeping bentgrass overexpressing Fld highlights the important role of this flavoprotein in protecting plants from multiple sources of adverse environmental conditions. The results obtained strongly suggest that using Fld, similar strategies could also be developed in important food crops to improve plant performance under adverse environmental conditions for enhanced agricultural production.

## Experimental procedures

### Plasmid construction

A 669‐bp DNA fragment containing the coding sequence of a pea FNR chloroplast‐targeting transit signal peptide (Newman and Gray, 1988; Serra *et al*., 1995; X12446) translationally fused to the cyanobacterial *Fld* gene (S68006) was chemically synthesized by Integrated DNA Technology (Coraville, IA) and used to produce a chimeric gene construct, pUbi:*FNR‐Fld*/p35S:*bar*. The vector contains the corn ubiquitin (Ubi) promoter driving *FNR‐Fld* and the cauliflower mosaic virus 35S (CaMV35S) promoter driving the *bar* gene for glufosinate (phosphinothricin) resistance as a selectable marker. The vector was delivered into the *A. tumefaciens* strain, LBA4404 for plant transformation.

### Plant transformation, propagation, maintenance and stress treatments

A commercial creeping bentgrass cultivar Penn A‐4 (supplied by HybriGene, Hubbard, OR) was employed for genetic transformation. The generation and nursing of TG plants were carried out as previously described (Luo *et al*., 2004a,b). The *FNR‐Fld* TG and WT control plants initially maintained in glasshouse were moved to a growth room with a 14‐h photoperiod for propagation. Both TG and WT control plants were clonally propagated from a single tiller and grown in cone‐tainers (4.0 × 20.3 cm; Dillen Products, Middlefield, OH), 4‐inch or 6‐inch pots (Dillen Products) using pure silica sand or commercial soil (Fafard 3‐B Mix, Fafard Inc., Anderson, SC). Shoots were trimmed weekly to maintain uniform plant growth. Growth room conditions were set up as previously described (Li *et al*., 2010).

To study plant growth and development, individual plants of both TG and WT controls were developed from a single tiller or 15 tillers for 12–22 weeks in cone‐tainers, 6‐inch or Elite 1200 pots (27.9 cm × 24.6 cm, Middlefield, OH) without/with clipping. To characterize vegetative‐to‐reproductive transition, 10‐week‐old unclipped plants were moved into a cold room for vernalization (8‐h photoperiod with a light supply of 120–170 μmol m^−2^ s^−1^ at 5 °C). After 30 weeks of continuous cold treatment, the grasses were transferred into a growth room with long‐day (LD) light regime (at 17–25 °C with a 16‐h photoperiod of 350–450 μmol m^−2^  s^−1^ light supply) for flowering.

For stress treatments, TG and WT control plants were propagated from stolons as previously described (Li *et al*., 2010). For water stress, three to eight replicates of both TG and WT control plants grown in trays (57 × 48 × 11 cm^3^) and in cone‐tainers with pure sand were maintained in growth room for 6–14 weeks. The plants were then subjected to drought stress by water withholding after a saturated watering.

For heat and oxidative stress treatments, four replicates of both TG and WT control plants grown in cone‐tainers with pure sand were maintained in growth room for 8 weeks. The plants were then subjected to heat stress as previously described (Li *et al*., 2013). The plant response to oxidative stress was assessed by daily spray of 30 μm of methyl viologen (MV, Sigma‐Aldrich Co. LLC) with 0.02% Triton X‐100 for 3 days.

To test the performance of WT and TG plants under different concentrations of N, five replicates of both TG and WT control plants were grown in cone‐tainers with pure sand and developed in growth room for 9 weeks. The plants and the sand were flushed using sufficient water to remove residual nutrients and then natured using modified 1× Murashige and Skoog (MS) solution supplemented with N at different concentrations (0, 0.4, 2, 10 or 40 mm). The preparation of MS solution and N addition was as previously described (Yuan *et al*., 2015). Three and five weeks after N starvation treatment, the shoots were harvested for further analysis to examine expression of the genes of interest and measure various physiological parameters. Plants were recovered from N stress by nurturing with 200‐ppm fertilizer and photographed for documentation.

### RNA isolation, cDNA synthesis, qPCR, RT‐PCR and northern blot

One hundred milligrams of young leaf tissues was employed for total RNA isolation using Trizol reagent (Invitrogen, Carlsbad, CA), and 2 μg of total RNAs was reverse‐transcribed using ProtoScript^®^ II Reverse Transcriptase (New England Biolabs, GA) following manufacturer's instructions. Fourfold diluted first‐strand cDNAs were stored at −20 °C for future use.

RT‐PCR for *FRN‐Fld* expression determination was conducted using the first‐strand cDNAs and gene‐specific primers (Table S1). qPCR was carried out on an iCycler iQ system (Bio‐Rad, Hercules, CA) in 25 μL of PCR solution containing 10 ppm SYBR Green I, 200 μm dNTPs, 1× PCR buffer, 1.5 mm MgCl_2_, Taq DNA polymerase and 40 nM each primer. There were three technical replicates for each of the three biological replicates. PCR was conducted with the following program: an initial DNA polymerase activation at 95 °C for 180 s followed by 40 cycles of 95 °C for 30 s, 60 °C for 20 s and 72 °C for 20 s. Finally, a melting curve was performed, and the PCR products were checked with 2% agarose gel in 0.5× TBE buffer with ethidium bromide. The ΔΔCt method was used for real‐time PCR analysis. Two reference genes, *AsACT1* and *AsUBQ* (Zhou *et al*., 2013), were used as endogenous controls. Relative expression level was calculated using the 2^−ΔΔCt^ formula. All primer pairs used for examining the expression levels of grass endogenous genes were designed based on the cloned creeping bentgrass cDNA sequences and listed in Table S1. For northern blot, a 513‐bp *Fld* gene fragment amplified from plasmid was used as probe. Probe was labelled with the [α‐^32^P] dCTP using the Prime‐It II Random Primer Labeling Kit (Stratagene, La Jolla, CA). RNA blot (10 μg of total RNAs) hybridizations were performed in Church buffer at 68°C. Hybridization signals were detected by exposure on a phosphor screen at room temperature overnight and scanning on a Typhoon 9400 phosphorimager (GE Healthcare Bio‐Sciences Corp., Piscataway, NJ).

### Measurement of leaf relative water content (RWC), electrolyte leakage (EL) and chlorophyll content

Plant leaf RWC, EL and total chlorophyll content were measured as previously described (Li *et al*., 2010).

### Measurement of reduced Trx content and NiR activities

Protein was extracted using Tris buffer of pH7.5 containing 100 mm Tris, 1 mm EDTA, 2 mm MgCl_2_, 20 mm DTT, 0.1 mm phenylmethanesulfonyl fluoride (PMSF). DTT and PMSF were added before use. Briefly, leaf tissues (0.1 g of fresh weight) from untreated and treated WT and TG plants were ground in ten volumes of ice‐cold extraction buffer and then centrifuged at 4 °C for 20 min (16 000***g***). The supernatants were transferred into new tubes and centrifuged again under the same conditions for another 20 min and then removed and kept on ice. Protein content was determined by a commercial Bradford assay (Bio‐Rad) using BSA as a standard following manufacturer's instruction.

The Trx assay was carried out following a previous protocol (Holmgren, 1979), the ratio of free Trx content under heat stress versus that under normal conditions was used to determine the electron transfer ability of TG and WT controls.

A spectrophotometric assay (Hagenman and Hucklesby, 1971; Losada and Paneque, 1971) was used to measure NiR activity. The reaction of NiR activity assay was conducted at 30 °C for 30 min followed by vigorously vortexing to stop the reaction. Each reaction mixture contained 0.3 mL of 0.5 M Tris buffer at pH 8.0, 0.2 mL of 20 mm potassium nitrite, 0.3 mL of 5 mm methyl viologen, 0.1 mL of crude enzyme, 0.3 mL of sodium dithionite solution (25 mg of sodium dithionite in 1 mL of 0.29 M NaHCO_3_) and 0.8 mL of H_2_O. After stopping the reaction, 1 mL each of Diazo‐coupling reagents was added to 2 mL of 100‐fold dilution of the reaction mixture, and the volume was made up to 5 mL with 1 mL of H_2_O. After 10 min, the optical density of the solution was determined at 540 nm. The nitrite content was calculated from a KNO_2_ standard curve. NiR activity was determined following Yuan *et al*. (2015).

### Statistical analysis

Summarized data (the counts, means and standard errors for each group) from three or more groups were subjected to a one‐way ANOVA and the Tukey's honestly significant difference *post hoc* tests. Means not sharing the same letter are statistically significantly different (*P* < 0.05).

## Conflict of interest

The authors declare no conflict of interests.

## Supporting information


**Figure S1** The *FNR‐Fld* fusion gene, its deduced protein sequences and overexpression in transgenic (TG) creeping bentgrass plants. (a) Synthesized nucleotide and deduced amino acid sequences of the FNR‐Fld fusion protein. The nucleotide sequence of the pea ferredoxin‐NADP^+^ reductase (FNR) chloroplast‐targeting transit signal peptide sequence was in capital letters and underlined. The flavodoxin (Fld) coding sequence was in lower case. The asterisk indicates the translation stop codon. (b) Schematic diagram of the *FNR‐Fld* chimeric gene expression construct, pUbi:*FNR‐Fld*/p35S:*bar*, in which the *FNR‐Fld* gene driven by the corn ubiquitin (Ubi) promoter was linked to the herbicide glufosinate (*phosphinothricin*) resistance gene, *bar*, driven by the cauliflower mosaic virus 35S (CaMV35S) promoter. (c) Integration and expression of the *FRN‐Fld* fusion gene in TG creeping bentgrass plants. Total RNA was extracted from young leaves of five representative TG lines. Transgene expression was determined by Northern hybridization using the *Fld* gene as a probe, and RT‐PCR on cDNA to amplify *Fld*. Total RNA and cDNA from wild type (WT) plants were used as negative controls. PCR products were fractionated on a 1.5% (w/v) agarose gel, stained with ethidium bromide. (d) *Fld* expression level in different TG lines was determined by dye‐based qPCR. Three biological replicates and three technical replicates were used for statistic analysis. Error bars indicate SD (n = 9). The statistically significant difference between groups was determined by one‐way ANOVA. Means not sharing the same letter are statistically significantly different (*P* < 0.05).
**Figure S2** Overexpression of Fld leads to modified plant growth and development in transgenic (TG) creeping bentgrass. (a) tiller numbers of the 22‐week‐old TG and wild type (WT) plants. The statistically significant difference between WT control and TG lines was determined by one‐way ANOVA. Means not sharing the same letter are statistically significantly different (*P* < 0.05). (b) characteristics of the inflorescence in Fld TG plants and WT controls. Student's *t*‐test was used to analyze the difference between the means from WT and TG line. Asterisks indicate a significant difference between WT and TG line: ***, *P *< 0.001.
**Figure S3** Transgenic (TG) turfgrass overexpressing flavodoxin (Fld) exhibits enhanced oxidative stress tolerance compared to wild type (WT) controls. TG and WT plants were re‐potted in cone‐tainers (TG4, 5 and 6) (a), or together in a big pot (TG4, 5, 6, 17 and 24) (b) and grown for 10 weeks under normal maintenance. Fully developed plants were sprayed daily with redox‐cycling herbicide, methyl viologen (MV, 30 μm with 0.02% Triton X‐100) for 3 days. The TG plants exhibited enhanced resistance to MV compared to WT controls. Photos were taken 8 and 11 days after the 3‐day MV treatments (a), or before, 4, 8 and 11 days after the 3‐day MV treatments (b).
**Figure S4** Transgenic (TG) turfgrass overexpressing flavodoxin (Fld) exhibits enhanced drought resistance compared to wild type (WT) controls. WT, TG4, 5, 6, 16, 17, 23 and 24 were repotted in cone‐tainers, and randomly set on the cone‐tainer rack for development for 10 weeks, followed by drought treatment. After 4 days water withholding, turfgrass tolerance was evaluated by visual estimation. WT plants were wilted, exhibiting higher sensitivity to water deprivation than transgenics.
**Figure S5** Estimation of gene expression using threshold cycles. Expression of housekeeping (*AsACT1* and *AsUBQ*) and *sHSP* genes were determined by Ct values collected from dye‐based qPCR with 3 ng of total RNA input. The expression levels of the three *sHSP* genes, *AsHSP17*,* AsHSP26.7* and *AsHSP26.8*, are all extremely low compared to the control housekeeping genes, *AsACT1* and *AsUBQ*, in both the TG and WT plants under normal growth conditions. The mean Ct values of the *AsHSP17*,* AsHSP26.7* and *AsHSP26.8* are 29.9, 33.9 and 37.4, respectively, whereas those of the *AsUBQ* and *AsACT1* are 22.9 and 21.4, respectively, in TG plants. Similarly, the Ct values of the *AsHSP17*,* AsHSP26.7* and *AsHSP26.8* are 32.8, 33.8 and 37.1, respectively, whereas those of the *AsUBQ* and *AsActin* are 23.1 and 21.1, respectively, in WT plants. The statistically significant difference between groups was determined by one‐way ANOVA. Means not sharing the same letter are statistically significantly different (*P* < 0.05).
**Figure S6** Flavodoxin (Fld)‐expressing transgenic (TG) plants exhibit enhanced tolerance to nitrogen (N) starvation. (a) Fully developed TG and wild type (WT) plants in cone‐tainers were maintained under normal growth conditions for 9 weeks and then treated with 1× MS medium containing 0, 0.4, 2, 10 or 40 mm nitrate (15 mL per day) for 5 weeks. (b) root development in WT and TG plants after 5 weeks of treatment with different N concentrations.
**Figure S7** Overexpression of flavodoxin (Fld) does not change plant response to salt stress. Wild type (WT) and two transgenic (TG) lines (TG4 and TG5) were repotted in cone‐tainers, and randomly set on the cone‐tainer rack for development for 10 weeks (a), followed by salinity treatment (200 mm NaCl) for 10 days (Li *et al*., 2010) (b). Specifically, the 10‐week‐old plants were watered daily with 10 mL of 200 ppm 20‐10‐20 fertilizer supplemented with 200 mm NaCl for 10 days. Plants were then recovered by watering daily with 200 ppm of water‐soluble fertilizer. Photos were taken before salt treatment (a) and 10 days after recovery (b).

**Table S1** Oligonucleotide sequences for gene specific amplification by PCR.
