# Supplementary material for: Ectopic expression of a cyanobacterial flavodoxin in creeping bentgrass impacts plant development and confers broad abiotic stress tolerance
Source: Plant Biotechnol J. 2016 Oct 20;15(4):433–46. doi: 10.1111/pbi.12638 (PMC5362689; doi:10.1111/pbi.12638)
Supplement: Supplementary file 1 — Figure S1 The FNR‐Fld fusion gene, its deduced protein sequences and overexpression in transgenic (TG) creeping bentgrass plants. (a) Synthesized nucleotide and deduced amino acid sequences of the FNR‐Fld fusion protein. The nucleotide sequence of the pea ferredoxin‐NADP+ reductase (FNR) chloroplast‐targeting transit signal peptide sequence was in capital letters and underlined. The flavodoxin (Fld) coding sequence was in lower case. The asterisk indicates the translation stop codon. (b) Schematic diagram of the FNR‐Fld chimeric gene expression construct, pUbi:FNR‐Fld/p35S:bar, in which the FNR‐Fld gene driven by the corn ubiquitin (Ubi) promoter was linked to the herbicide glufosinate (phosphinothricin) resistance gene, bar, driven by the cauliflower mosaic virus 35S (CaMV35S) promoter. (c) Integration and expression of the FRN‐Fld fusion gene in TG creeping bentgrass plants. Total RNA was extracted from young leaves of five representative TG lines. Transgene expression was determined by Northern hybridization using the Fld gene as a probe, and RT‐PCR on cDNA to amplify Fld. Total RNA and cDNA from wild type (WT) plants were used as negative controls. PCR products were fractionated on a 1.5% (w/v) agarose gel, stained with ethidium bromide. (d) Fld expression level in different TG lines was determined by dye‐based qPCR. Three biological replicates and three technical replicates were used for statistic analysis. Error bars indicate SD (n = 9). The statistically significant difference between groups was determined by one‐way ANOVA. Means not sharing the same letter are statistically significantly different (P < 0.05). Figure S2 Overexpression of Fld leads to modified plant growth and development in transgenic (TG) creeping bentgrass. (a) tiller numbers of the 22‐week‐old TG and wild type (WT) plants. The statistically significant difference between WT control and TG lines was determined by one‐way ANOVA. Means not sharing the same letter are statistically significant [file PBI-15-433-s001.pptx]

## Slide 1
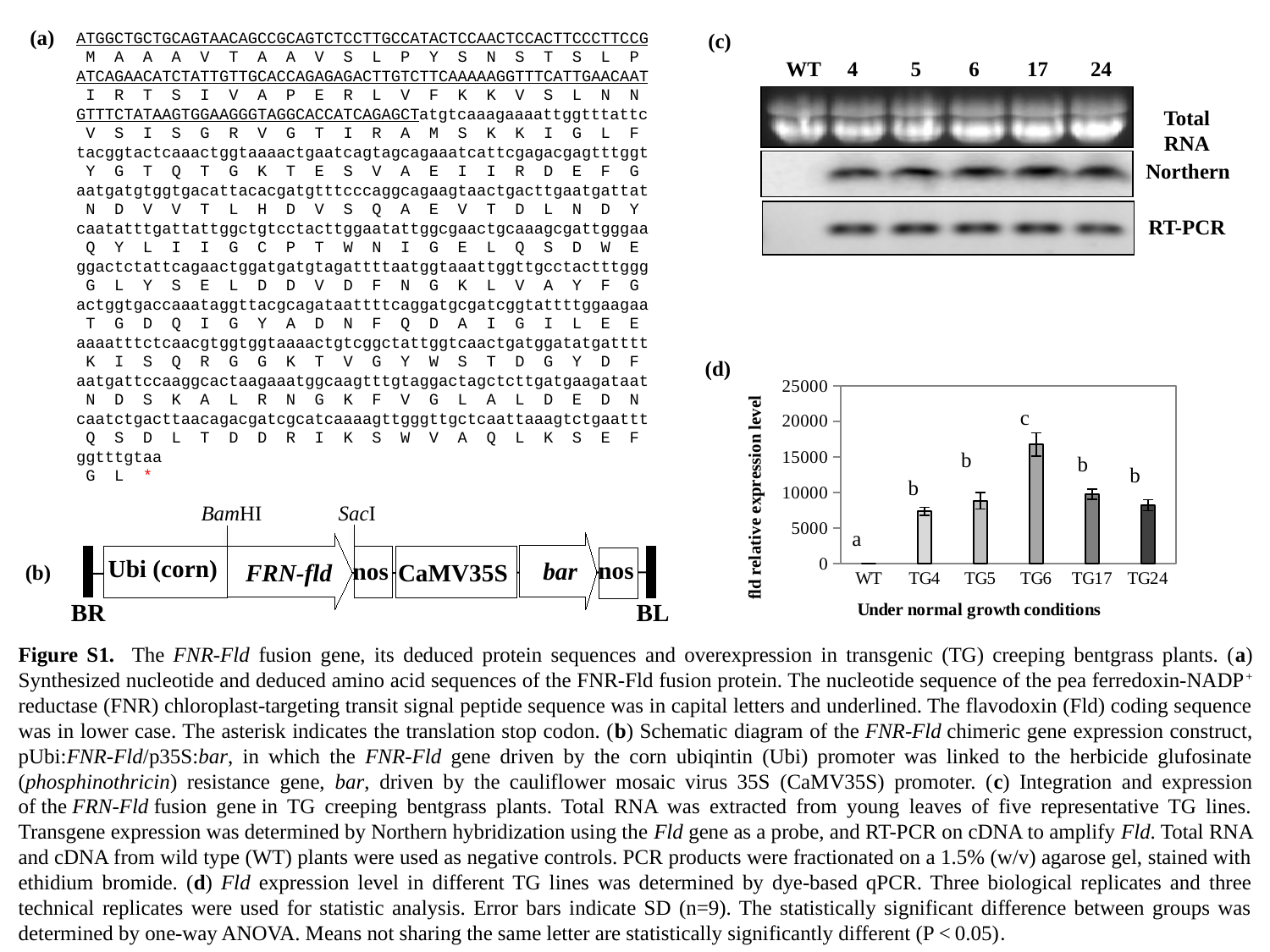

(a)
ATGGCTGCTGCAGTAACAGCCGCAGTCTCCTTGCCATACTCCAACTCCACTTCCCTTCCG
 M A A A V T A A V S L P Y S N S T S L P
ATCAGAACATCTATTGTTGCACCAGAGAGACTTGTCTTCAAAAAGGTTTCATTGAACAAT
 I R T S I V A P E R L V F K K V S L N N
GTTTCTATAAGTGGAAGGGTAGGCACCATCAGAGCTatgtcaaagaaaattggtttattc
 V S I S G R V G T I R A M S K K I G L F
tacggtactcaaactggtaaaactgaatcagtagcagaaatcattcgagacgagtttggt
 Y G T Q T G K T E S V A E I I R D E F G
aatgatgtggtgacattacacgatgtttcccaggcagaagtaactgacttgaatgattat
 N D V V T L H D V S Q A E V T D L N D Y
caatatttgattattggctgtcctacttggaatattggcgaactgcaaagcgattgggaa
 Q Y L I I G C P T W N I G E L Q S D W E
ggactctattcagaactggatgatgtagattttaatggtaaattggttgcctactttggg
 G L Y S E L D D V D F N G K L V A Y F G
actggtgaccaaataggttacgcagataattttcaggatgcgatcggtattttggaagaa
 T G D Q I G Y A D N F Q D A I G I L E E
aaaatttctcaacgtggtggtaaaactgtcggctattggtcaactgatggatatgatttt
 K I S Q R G G K T V G Y W S T D G Y D F
aatgattccaaggcactaagaaatggcaagtttgtaggactagctcttgatgaagataat
 N D S K A L R N G K F V G L A L D E D N
caatctgacttaacagacgatcgcatcaaaagttgggttgctcaattaaagtctgaattt
 Q S D L T D D R I K S W V A Q L K S E F
ggtttgtaa
 G L *
(c)
WT 4 5 6 17 24
Total RNA
Northern
RT-PCR
(d)
### Chart
| Category | |
|---|---|
| WT | 1.0 |
| TG4 | 7359.35067800751 |
| TG5 | 8847.90622177135 |
| TG6 | 16749.4230645829 |
| TG17 | 9786.548986874575 |
| TG24 | 8219.445787768655 |c
b
b
b
b
a
BamHI
SacI
Ubi (corn)
nos
nos
bar
FRN-fld
CaMV35S
BL
BR
(b)
Figure S1. The FNR-Fld fusion gene, its deduced protein sequences and overexpression in transgenic (TG) creeping bentgrass plants. (a) Synthesized nucleotide and deduced amino acid sequences of the FNR-Fld fusion protein. The nucleotide sequence of the pea ferredoxin-NADP+ reductase (FNR) chloroplast-targeting transit signal peptide sequence was in capital letters and underlined. The flavodoxin (Fld) coding sequence was in lower case. The asterisk indicates the translation stop codon. (b) Schematic diagram of the FNR-Fld chimeric gene expression construct, pUbi:FNR-Fld/p35S:bar, in which the FNR-Fld gene driven by the corn ubiqintin (Ubi) promoter was linked to the herbicide glufosinate (phosphinothricin) resistance gene, bar, driven by the cauliflower mosaic virus 35S (CaMV35S) promoter. (c) Integration and expression of the FRN-Fld fusion gene in TG creeping bentgrass plants. Total RNA was extracted from young leaves of five representative TG lines. Transgene expression was determined by Northern hybridization using the Fld gene as a probe, and RT-PCR on cDNA to amplify Fld. Total RNA and cDNA from wild type (WT) plants were used as negative controls. PCR products were fractionated on a 1.5% (w/v) agarose gel, stained with ethidium bromide. (d) Fld expression level in different TG lines was determined by dye-based qPCR. Three biological replicates and three technical replicates were used for statistic analysis. Error bars indicate SD (n=9). The statistically significant difference between groups was determined by one-way ANOVA. Means not sharing the same letter are statistically significantly different (P < 0.05).

## Slide 2
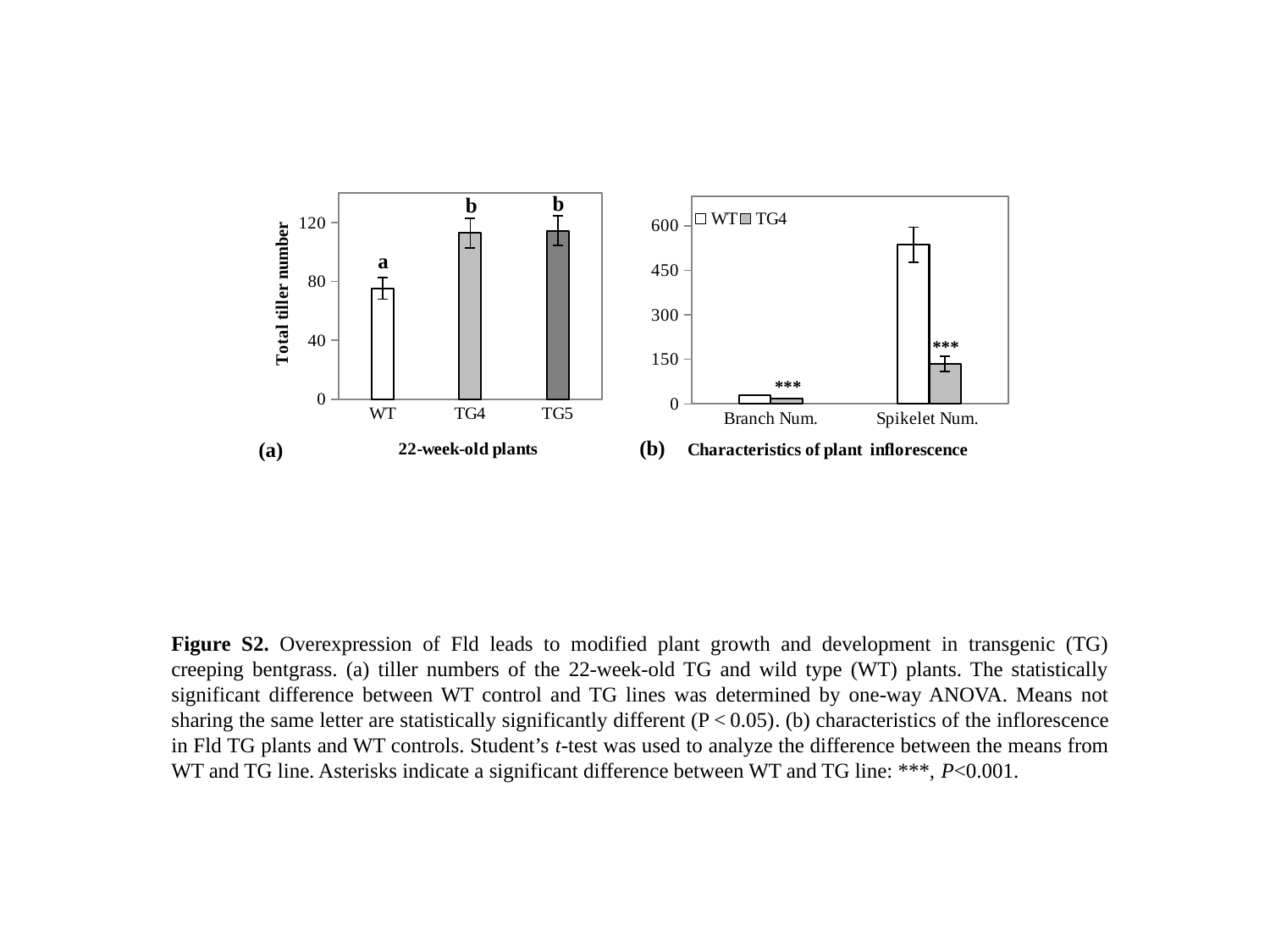

### Chart
| Category | |
|---|---|
| WT | 75.3333333333332 |
| TG4 | 113.0 |
| TG5 | 114.5 |b
b
a
(a)
### Chart
| Category | WT | TG4 |
|---|---|---|
| Branch Num. | 28.875 | 18.0 |
| Spikelet Num. | 535.8 | 134.2 |***
***
 (b)
Figure S2. Overexpression of Fld leads to modified plant growth and development in transgenic (TG) creeping bentgrass. (a) tiller numbers of the 22-week-old TG and wild type (WT) plants. The statistically significant difference between WT control and TG lines was determined by one-way ANOVA. Means not sharing the same letter are statistically significantly different (P < 0.05). (b) characteristics of the inflorescence in Fld TG plants and WT controls. Student’s t-test was used to analyze the difference between the means from WT and TG line. Asterisks indicate a significant difference between WT and TG line: ***, P<0.001.

## Slide 3
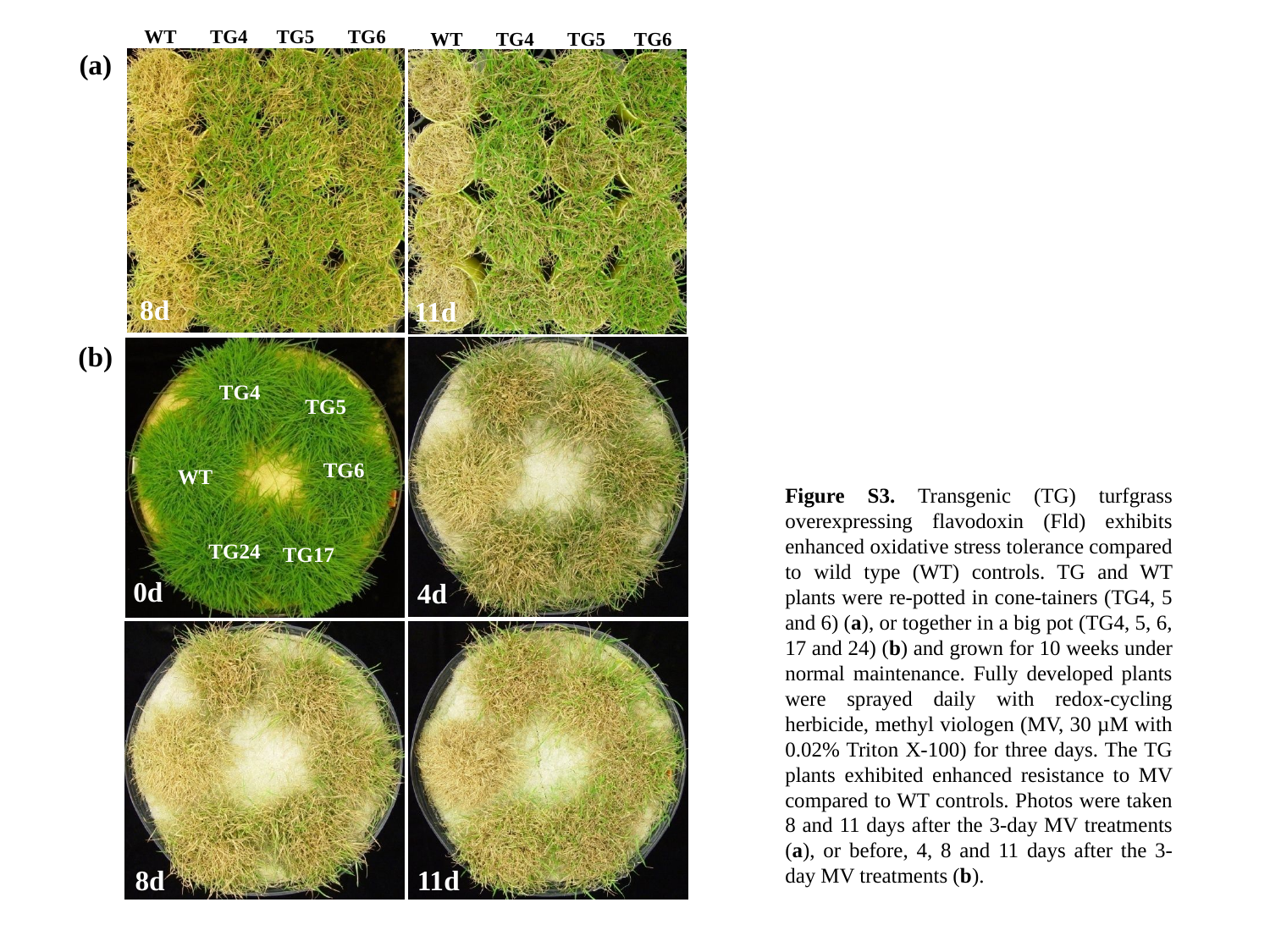

WT TG4 TG5 TG6
 WT TG4 TG5 TG6
8d
11d
(a)
(b)
(b)
0d
TG4
TG5
TG6
WT
TG24
TG17
4d
8d
11d
Figure S3. Transgenic (TG) turfgrass overexpressing flavodoxin (Fld) exhibits enhanced oxidative stress tolerance compared to wild type (WT) controls. TG and WT plants were re-potted in cone-tainers (TG4, 5 and 6) (a), or together in a big pot (TG4, 5, 6, 17 and 24) (b) and grown for 10 weeks under normal maintenance. Fully developed plants were sprayed daily with redox-cycling herbicide, methyl viologen (MV, 30 µM with 0.02% Triton X-100) for three days. The TG plants exhibited enhanced resistance to MV compared to WT controls. Photos were taken 8 and 11 days after the 3-day MV treatments (a), or before, 4, 8 and 11 days after the 3-day MV treatments (b).

## Slide 4
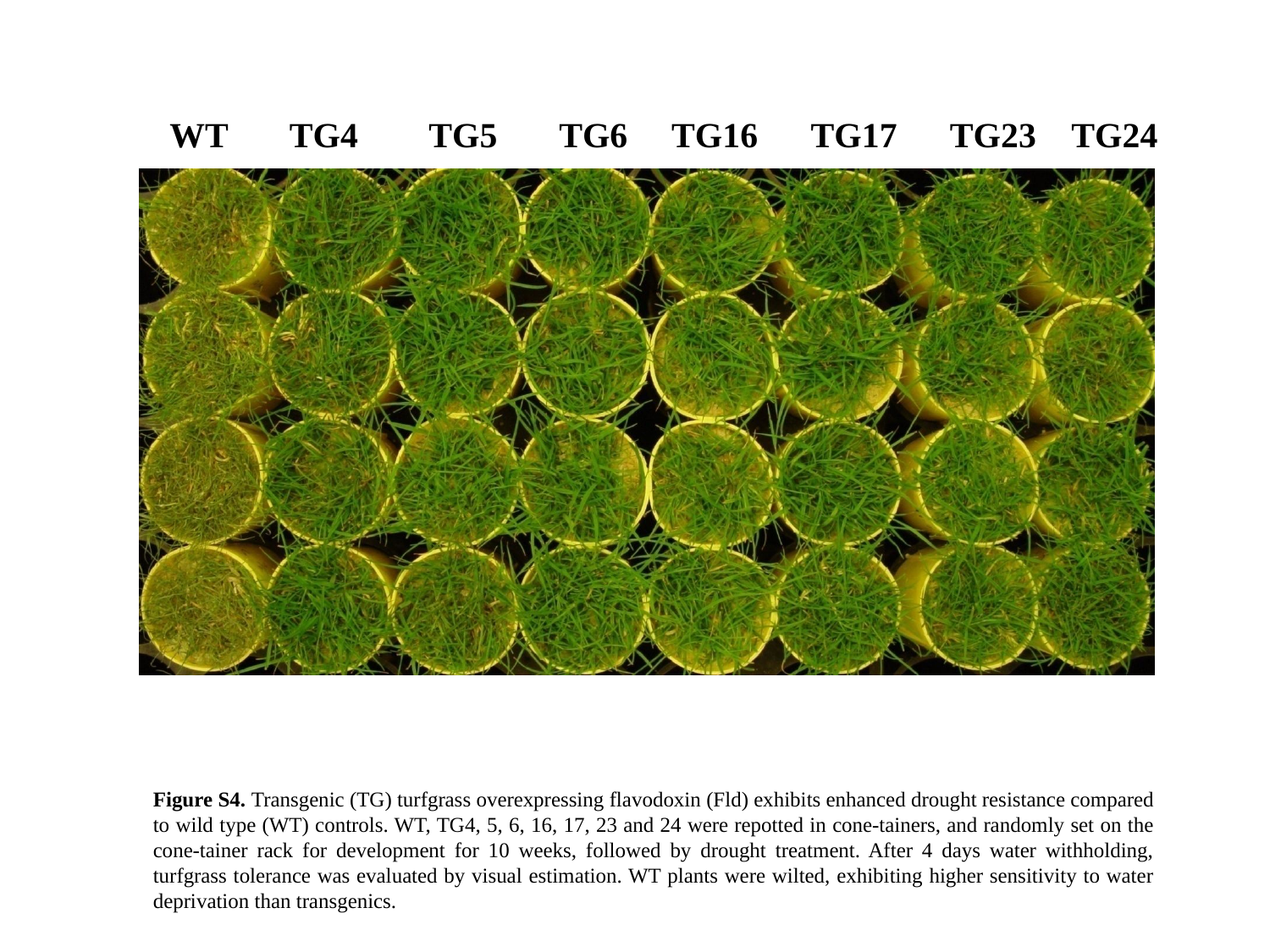

WT TG4 TG5 TG6 TG16 TG17 TG23 TG24
Figure S4. Transgenic (TG) turfgrass overexpressing flavodoxin (Fld) exhibits enhanced drought resistance compared to wild type (WT) controls. WT, TG4, 5, 6, 16, 17, 23 and 24 were repotted in cone-tainers, and randomly set on the cone-tainer rack for development for 10 weeks, followed by drought treatment. After 4 days water withholding, turfgrass tolerance was evaluated by visual estimation. WT plants were wilted, exhibiting higher sensitivity to water deprivation than transgenics.

## Slide 5
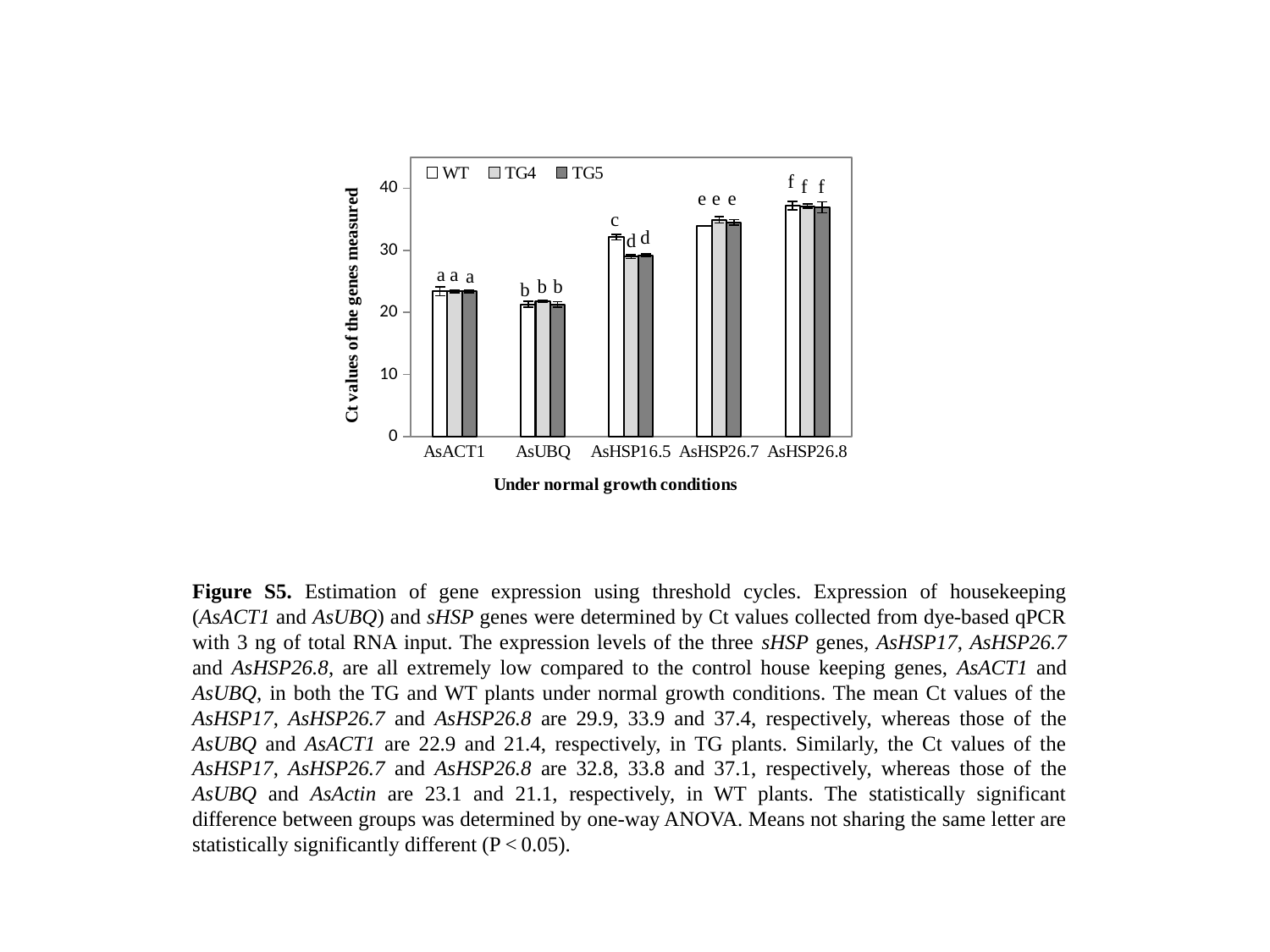

### Chart
| Category | WT | TG4 | TG5 |
|---|---|---|---|
| AsACT1 | 23.4 | 23.4 | 23.4 |
| AsUBQ | 21.3 | 21.8 | 21.3 |
| AsHSP16.5 | 32.1 | 29.0 | 29.2 |
| AsHSP26.7 | 33.9 | 34.9 | 34.5 |
| AsHSP26.8 | 37.2 | 37.1 | 36.9 |f
f
f
e
e
e
c
d
d
a
a
a
b
b
b
Figure S5. Estimation of gene expression using threshold cycles. Expression of housekeeping (AsACT1 and AsUBQ) and sHSP genes were determined by Ct values collected from dye-based qPCR with 3 ng of total RNA input. The expression levels of the three sHSP genes, AsHSP17, AsHSP26.7 and AsHSP26.8, are all extremely low compared to the control house keeping genes, AsACT1 and AsUBQ, in both the TG and WT plants under normal growth conditions. The mean Ct values of the AsHSP17, AsHSP26.7 and AsHSP26.8 are 29.9, 33.9 and 37.4, respectively, whereas those of the AsUBQ and AsACT1 are 22.9 and 21.4, respectively, in TG plants. Similarly, the Ct values of the AsHSP17, AsHSP26.7 and AsHSP26.8 are 32.8, 33.8 and 37.1, respectively, whereas those of the AsUBQ and AsActin are 23.1 and 21.1, respectively, in WT plants. The statistically significant difference between groups was determined by one-way ANOVA. Means not sharing the same letter are statistically significantly different (P < 0.05).

## Slide 6
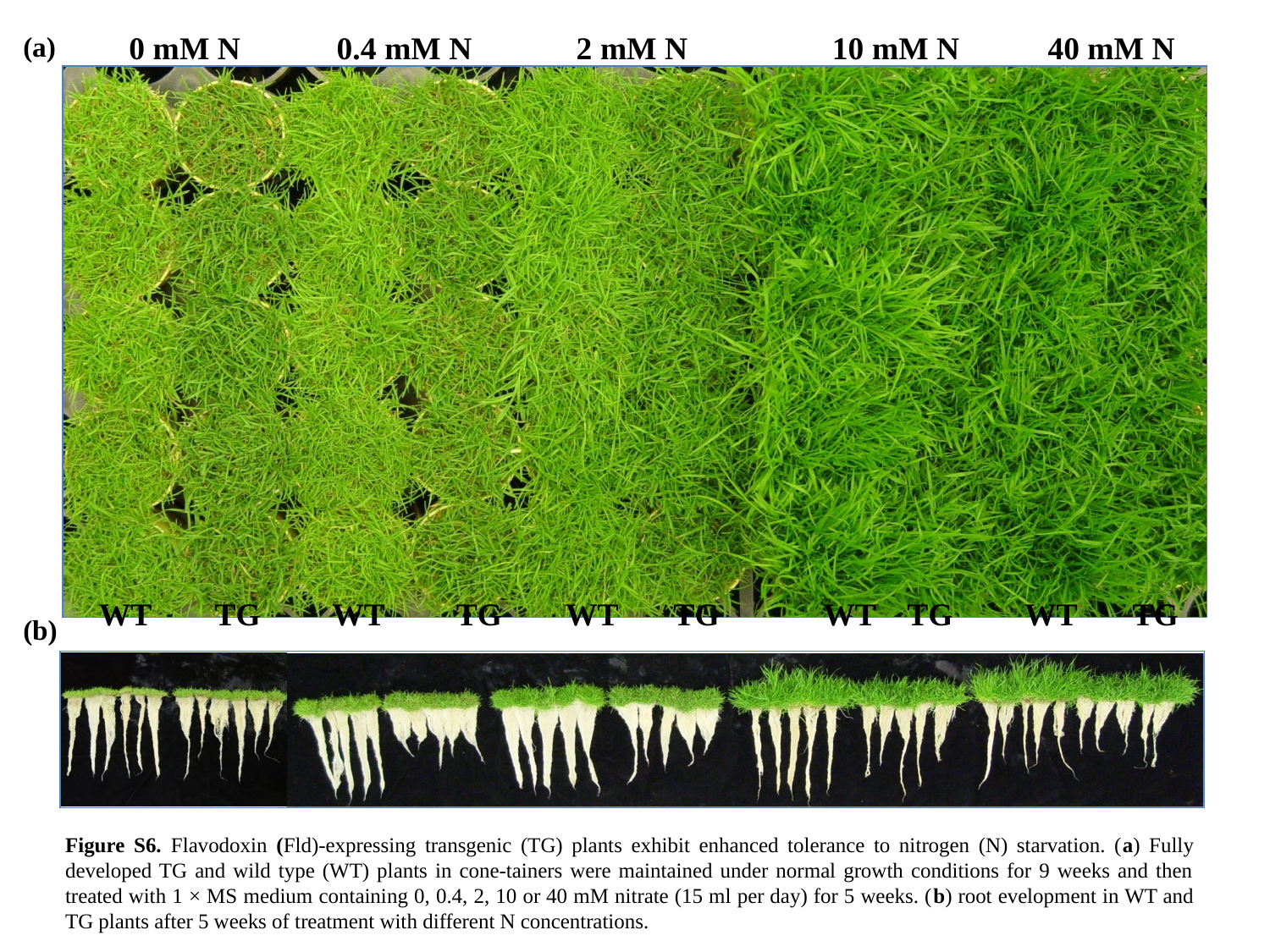

0 mM N 0.4 mM N 2 mM N 10 mM N 40 mM N
(a)
(b)
 WT TG WT TG WT TG WT TG WT TG
Figure S6. Flavodoxin (Fld)-expressing transgenic (TG) plants exhibit enhanced tolerance to nitrogen (N) starvation. (a) Fully developed TG and wild type (WT) plants in cone-tainers were maintained under normal growth conditions for 9 weeks and then treated with 1 × MS medium containing 0, 0.4, 2, 10 or 40 mM nitrate (15 ml per day) for 5 weeks. (b) root evelopment in WT and TG plants after 5 weeks of treatment with different N concentrations.

## Slide 7
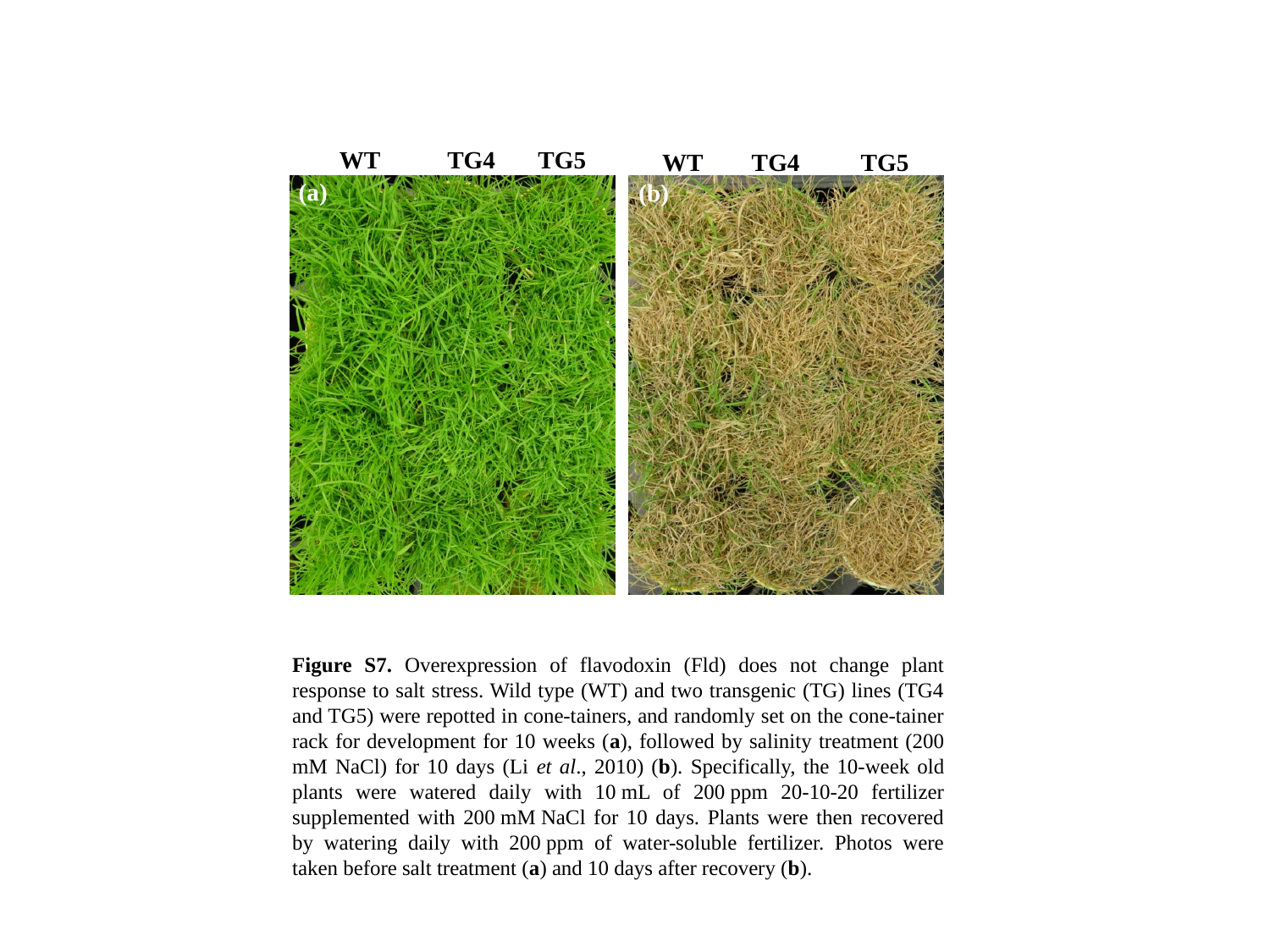

WT TG4 TG5
WT TG4 TG5
(a)
(b)
Figure S7. Overexpression of flavodoxin (Fld) does not change plant response to salt stress. Wild type (WT) and two transgenic (TG) lines (TG4 and TG5) were repotted in cone-tainers, and randomly set on the cone-tainer rack for development for 10 weeks (a), followed by salinity treatment (200 mM NaCl) for 10 days (Li et al., 2010) (b). Specifically, the 10-week old plants were watered daily with 10 mL of 200 ppm 20-10-20 fertilizer supplemented with 200 mM NaCl for 10 days. Plants were then recovered by watering daily with 200 ppm of water-soluble fertilizer. Photos were taken before salt treatment (a) and 10 days after recovery (b).
