# Supplementary material for: Ectopic expression of a cyanobacterial flavodoxin in creeping bentgrass impacts plant development and confers broad abiotic stress tolerance
Source: Plant Biotechnol J. 2016 Oct 20;15(4):433–46. doi: 10.1111/pbi.12638 (PMC5362689; doi:10.1111/pbi.12638)
Supplement: Supplementary file 2 — Table S1 Oligonucleotide sequences for gene specific amplification by PCR. [file PBI-15-433-s002.docx]

**Supplementary Materials**

**Table S1**  Oligonucleotide sequences for gene specific amplification by PCR/qPCR

| Gene name | Accession number | Oligonucleotide | Predicted PCR product size (bp) | |
| --- | --- | --- | --- | --- |
|  |  |  | cDNA | Genomic DNA |
| FRN-fld | S68006 | F-ATGTCAAAGAAAATTGGTTTATTC | 513 | 513 |
|  |  | R-TTACAAACCAAATTCAGAC |  |  |
| Fld | S68006 | F-TTCAGGATGCGATCGGTATTT | 119 | 119 |
|  |  | R-CTTGCCATTTCTTAGTGCCTTG |  |  |
| AsUBQ | JX570760 | F- GACAACGTCAAGGCCAAGAT | 264 | n/a |
|  |  | R- GGCGTCATCGACCTTGTAGA |  |  |
| AsActin | JX644005 | F- TCACTGAGGCTCCTTTGAACCCAA | 241 | n/a |
|  |  | R- AGATCCCGACCAGCAAGATCCAA |  |  |
| * AsNCA | [KT803721](http://www.ncbi.nlm.nih.gov/nuccore/KT803721) | F- GGGAGCAGGGAGTGGTACTT | 170 | n/a |
|  |  | R- GACGAGCGCCTTCTTGATGC |  |  |
| *AsPR1 | [KT803722](http://www.ncbi.nlm.nih.gov/nuccore/KT803722) | F- CCACACCACCTGCGTGTAGTG | 145 | n/a |
|  |  | R- GAGAACATCTTCTGGGGCTCC |  |  |
| *AsACP450 | KT803719 | F- CTACAAGATCATTGCCCAGGAGGT | 137 | n/a |
|  |  | R- CGAAGGAGATCTTGCAGATGGTGT |  |  |
| *AsHSP17.0 | [KT272405](http://www.ncbi.nlm.nih.gov/nucleotide/961349973?report=genbank&log$=nuclalign&blast_rank=1&RID=J1G6SRAG01R) | F-AAGGTGGAGGTCGAGGAT | 124 | n/a |
|  |  | R- TGAAGCGCCTGACAAACT |  |  |
| *AsHSP26.7 | [KT803715](http://www.ncbi.nlm.nih.gov/nuccore/KT803715) | F-CCCACAACACGTCGTTCA | 90 | n/a |
|  |  | R-CCGCATCTTCACCTCCTTATC |  |  |
| *AsHSP26.8 | [KT803716](http://www.ncbi.nlm.nih.gov/nuccore/KT803716) | F- CCCACGACACGCCGTTCG | 90 | n/a |
|  |  | R- CCGCATCTTCACCTCCTTTTC |  |  |
| *AsNiR | [KR911829](http://www.ncbi.nlm.nih.gov/nucleotide/910276098?report=genbank&log$=nuclalign&blast_rank=1&RID=J1GN68G9014) | F- CCTACACCAACCTTCTCTCCGC | 172 | n/a |
|  |  | R- CCGAACTTGCCGTCCTTCTCC |  |  |
| *AsTrx | Unpublished | F- GAACGCCTCCAAGCTGATGGT | 180 | n/a |
|  |  | F- GTGGCCGTCATGATACTCTTC |  |  |
| #AsNRT2 | [KT803712](http://www.ncbi.nlm.nih.gov/nuccore/KT803712) | F- GTTCTGGATGAGCTCCATGTT | 362 | n/a |
|  |  | R- CGAAGCAGTAGCCGTATGTTAG |  |  |

Note: *The listed primer pairs were designed from the degenerated primers amplified, cloned and sequenced cDNA sequences of creeping bentgrass. #: The primer pairs were designed based on three sequences AS31755, AS31756, AS31758 which are from RNAseq database (unpublished).
